# Timing of molt of barn swallows is delayed in a rare *Clock* genotype

**DOI:** 10.7717/peerj.17

**Published:** 2013-02-12

**Authors:** Nicola Saino, Maria Romano, Manuela Caprioli, Mauro Fasola, Roberto Lardelli, Pierfrancesco Micheloni, Chiara Scandolara, Diego Rubolini, Luca Gianfranceschi

**Affiliations:** 1 Department of Biosciences, University of Milan, Milan, Italy; 2 Dipartimento di Scienze della Terra e dell’Ambiente, Pavia, Italy; 3 Swiss Ornithological Institute, Sempach, Switzerland; 4 Istituto Superiore per la Protezione e per la Ricerca Ambientale (ISPRA), Ozzano Emilia (BO), Italy

**Keywords:** Plumage molt, Genetic control, *Clock* gene, Hirundo rustica, Migration, Sexual dimorphism, Molecular sexing

## Abstract

Photoperiodic responses are major factors entraining circannual life-cycles, functioning to adaptively synchronize annual routines to seasonal fluctuations in ecological conditions. Photoperiodism in physiology and behaviour is enforced by genes, including the vertebrate *Clock* orthologues, which are associated, for example, with phenology of migration in fish and breeding in birds. However, the role of *Clock* in photoperiodic plumage molt processes is unknown. We analyzed variation in molt schedules in relation to *Clock* genotype, using the long-distance migratory barn swallow (*Hirundo rustica*) as a model and by identifying males and females using molecular sexing techniques. Consistently with previous studies, we found one very common (*Q*_7_) and two rare (*Q*_6_, *Q*_8_) variants of a functionally significant *Clock* polyglutamine repeat. Molt schedules of primary wing feathers of swallows during their wintering period in Nigeria differed among *Clock* genotypes: rare (1.1%) *Q*_7_/*Q*_8_ heterozygotes had significantly delayed molt compared to the other genotypes. Molt schedules did not differ between males and females, and no differential association between molt and *Clock* in relation to sex emerged. The same rare *Clock* genotype that exhibited delayed breeding in Europe was here found to delay molt in Africa. Though based on a limited number of *Q*_7_/*Q*_8_ individuals from an otherwise very large sample, these novel results suggest that *Clock* is involved in the photoperiodic control of both molt and breeding, potentially also via reciprocal carry-over effects. If confirmed in species with higher *Clock* polymorphism, present results may have far-reaching consequences for the study of photoperiodic control of molt and expression of annual routines.

## Introduction

Organisms are selected to match the timing of life stages to seasonal variation in ecological conditions. A major extrinsic driver of synchronization of phenological or physiological events, such as breeding or migration, to seasonal fluctuations in environmental conditions is variation in photoperiod (Gwinner, 1986; Beacham & Murray, 1988; Bradshaw & Holzapfel, 2001; Dawson et al., 2001; Goldman, 2001; Gwinner, 2003). Rhythmicity of circannual life-cycles can be enforced by so called “circadian clocks” that can sense variation in day/night cycles and cause shifts in physiology and behaviour by triggering a cascade of photoperiodic responses (Darlington et al., 1998; Yasuo et al., 2003; Kyriacou et al., 2007). Genes responsible for regulation of such circadian biochemical oscillators are phylogenetically highly conserved and their mechanism of action has been well described (Bell-Pedersen et al., 2005). *Clock* genes, in particular, seem to play a role in the photoperiodic responses of diverse invertebrates and vertebrates, including migration in fish and breeding in birds (DeBruyne et al., 2006; O’Malley & Banks, 2008; Liedvogel et al., 2009), but the studies that have investigated photoperiodism in relation to *Clock* in the wild are still sparse.

A domain of *Clock* genes which is functionally relevant to photoperiodism is the carboxyl-terminal polyglutamine repeat (Poly-Q), as the number of Poly-Q repeats is known to affect circadian rhythms in mice (Gekakis et al., 1998; Vitaterna et al., 2006). In the Chinhook salmon (*Onchorhynchus tshawytscha*) latitudinal variation in *Clock* allele frequencies parallels that of spring migration phenology (O’Malley & Banks, 2008; O’Malley, Ford & Hard, 2010). Among birds, timing of breeding of the resident/short-distance migratory blue tit (*Cyanistes* *caeruleus*) is associated with *Clock* polymorphism and a cline in *Clk*polyQcds allele frequency exists whereby the number of Poly-Q repeats increases with latitude (Johnsen et al., 2007; Liedvogel et al., 2009). In the long-distance migratory barn swallow (*Hirundo rustica*) breeding date is also predicted by *Clock* genotype (Caprioli et al., 2012). In all these instances, late phenology has been found to be associated with an increase in the number of Poly-Q *Clock* terminal repeats. However, other studies of birds (e.g. on great tits, *Parus major*; swallows of the genus *Tachycineta*) have found very low polymorphism at *Clock* and no relationship between *Clock* and breeding phenology (Liedvogel & Sheldon, 2010; Dor et al., 2012). As huge variation in *Clock* polymorphism exists even among closely related species (e.g. *Tachycineta* swallow; blue tit and great tit) (Liedvogel et al., 2009; Liedvogel & Sheldon, 2010; Caprioli et al., 2012; Dor et al., 2012), difficulties in identifying an association between phenology and *Clock* may in some species stem from rarity of phenologically deviant, potentially counter-selected genotypes, in combination with large environmental “noise” in breeding phenological data.

It has been suggested that photoperiod may have no or minor role in synchronizing circannual rhythms at the tropics where limited oscillations in photoperiod occur through the year (e.g. Miller, 1965; Cockrem, 1995; see Hau, Wikelski & Wingfield, 1998). On the other hand, even minor changes in photoperiod can be sensed by tropical birds and cause for example gonadal maturation (Hau, Wikelski & Wingfield, 1998; see also Burns, 1985; Wayne & Rissman, 1991 for effects of tropical photoperiod on other vertebrates), suggesting that photoperiod can act as a *zeitgeber* of circannual rhythms also at low latitudes. In addition, at the tropics information from daytime light intensity may be integrated with that of daylength or time of sunrise and sunset to serve as a circannual *zeitgeber* (Gwinner & Scheuerlein, 1998; Gwinner, 2003; Goymann et al., 2012; see also Bentley et al., 1998). However, we are aware of no study where the association between *Clock* and photoperiodic events has been analyzed at the tropics in vertebrates.

Annual life-cycles are often cadenced by a regular succession of fundamental activities which can be reciprocally constrained by time and/or resource limitation (McNamara & Houston, 2008). The evolution and expression of individual photoperiodic traits therefore occur under the limits set by competing activities. For example, during any single year, migratory birds must switch from breeding to plumage molting and migration, which are all highly demanding activities, both in terms of resources and time (Newton, 2008; Newton, 2011). Although in some cases it may be enforced by time constraints (see Hemborg & Lundberg, 1998; Hemborg, 1999; Schaub & Jenni, 2000), overlap between molt and migration or breeding can be detrimental to individual performance and viability because of costliness or direct negative reciprocal interference of these activities, as is the case when molt of wing feathers impairs flight performance and thus migration (Ginn & Melville, 1983; Lindström, Visser & Daan, 1993; Jenni & Winkler, 1994; Berthold, 1996; Murphy, 1996; Norris et al., 2004; Newton, 2008). As a result of such reciprocal constraints, any shift in timing of these activities can carry-over on the following events in the annual routine (McNamara & Houston, 2008; Harrison et al., 2011). Hence, an association between timing of any specific activity and genotype may arise either as a direct genetic effect on that particular activity or as a consequence of a constraint, set by a genetic effect on a different activity, and mediated by limiting availability of time or resources.

Circannual rhythmicity of breeding, migration and molt in birds is at least partly under the control of photoperiod (Gwinner, 1986; Gwinner, 2003; Sharp, 2006). The timing and speed of plumage molt, in particular, has been shown to be controlled by photoperiod in a number of studies of diverse species, particularly from temperate latitudes (Dawson et al., 2001; Gwinner, 2003; Dawson, 2004; Griggio et al., 2009; Vágási, Pap & Barta, 2010). Importantly, large heritability estimates of timing of molt in birds have been found, implying large genetic variance in this trait at least in the few species that have been studied so far (Larsson, 1996; Helm & Gwinner, 1999). To the best of our knowledge, however, no study has directly analyzed the association between variation in timing of photoperiodic molt processes and *Clock*, which is known to be a major mechanism mediating photoperiodic responses.

In this study of barn swallows (*Hirundo rustica*), our aim thus was to start filling this gap of knowledge by analyzing the progress of molt in the African winter quarters in free-living individuals that were subsequently genotyped at *Clock* and sexed using molecular techniques. We expected *Clock* polymorphism to be low (Dor et al., 2011; Caprioli et al., 2012). Because female barn swallows with a rare (*Q*_7_/*Q*_8_) genotype were found to have delayed breeding (Caprioli et al., 2012) and, in general, relatively “long alleles” (e.g. *Q*_8_) are expected to be associated with delayed photoperiodic activities (see above), we had the directional prediction that *Q*_7_/*Q*_8_ individuals delayed molt relative to the other genotypes. This was expected either because of consistently delayed photoperiodic response by this genotype in both breeding and molt, or because of carry-over effects of timing of breeding on timing of molt (or vice versa), given the presumably tight annual routines of this long-distance migrant that has up to three breeding attempts during a single breeding season and may take up to 135 days to complete molt (Jenni & Winkler, 1994; Møller, 1994; Cramp, 1998; Turner, 2006; Altwegg et al., 2012).

Because the natural and sexual selection forces acting on males or females can differ, sex is an additional factor expected to affect circannual routines, and thus sex-specific phenology in e.g. migration or molt (Morbey & Ydenberg, 2001; Kissner, Weatherhead & Francis, 2003; Saino et al., 2010; Spottiswoode & Saino, 2010). Thus, in a companion study (Saino et al., 2013) we investigated variation in timing of molt and showed that no difference in timing of molt between males and females exists for wing, tail or crown feathers.

## Materials and methods

### Study organism

The barn swallow is a small (ca. 18 g), long-distance migratory, aerially insectivorous passerine bird. Individuals breeding in Europe winter in sub-Saharan Africa, exhibiting significant migratory connectivity (i.e. individuals breeding in the same geographical area tend also to winter in the same area) (Møller, 1994; Cramp, 1998; Ambrosini, Møller & Saino, 2009). A single annual molt of remiges (wing feathers) and rectrices (tail feathers) occurs, mostly in the African winter quarters (Ginn & Melville, 1983; Cramp, 1998; van den Brink, Bijlsma & van der Have, 2000; Turner, 2006; Jenni & Winkler, 1994; Altwegg et al., 2012). Primary wing feathers, which are at the focus of the present study because they are the most aerodynamically important, are molted sequentially starting from the innermost one (P1). Barn swallows start arriving in Europe by mid-March but late individuals can arrive as late as May–June. Females tend to arrive later than males but the difference in arrival date is small (ca. 5 days) (Møller, 1994). Autumn migration in Europe mostly occurs in September–October.

Low polymorphism has been recorded at *Clock* in seven breeding barn swallow populations (Dor et al., 2011; Caprioli et al., 2012; N Saino, M Caprioli & AP Møller, unpublished data). Yet, in an Italian population where the association between *Clock* and breeding phenology has been investigated, females with a rare (*Q*_7_/*Q*_8_) genotype have been observed to delay breeding, with negative consequence on seasonal reproductive success. In addition, frequency of *Q*_7_/*Q*_8_ is higher among yearlings than among two or more years old individuals, suggesting that *Clock* affects breeding phenology in this species and selection occurs against a rare, phenologically deviant genotype (Caprioli et al., 2012).

### Field methods

We captured barn swallows on 12–16 February 2012 at a nocturnal roosting site in the remote village of Boje, eastern Nigeria (6°17’26” N, 8°55’51”E) (see also Saino et al., 2013). This site is a traditional roosting place of hundreds of thousands of barn swallows wintering in the area at least since 1996 (P Micheloni, unpublished data), but likely much earlier times. Ring recoveries have shown that this wintering area receives swallows originating from or breeding in at least twelve, mostly western or central European countries. Sampling at a roost to study molt of barn swallows and its temporal change has also been adopted in previous studies (Møller et al., 1995; Møller et al., 2011; van den Brink, Bijlsma & van der Have, 2000).

To get a picture of the association between molt and *Clock* genotype around completion of molt of the primary wing feathers (hereafter “primaries”) shortly before spring migration, we decided to conduct the study in mid-February, when 40%–50% of the birds have normally completed molt (P Micheloni, unpublished data). However, we also aimed at retaining variation in molt stages in the sample and we therefore a priori decided to collect a sample composed for 3/4 by birds that were still actively molting their wing feathers and for 1/4 by birds that had completed molt. Individuals within these categories were then sampled randomly. For the purposes of this study, each primary (excluding the vestigial P10; P1–P9 were counted outwards, so that P1 was the innermost of the 10 primaries) was scored for molt stage using a standard 6 levels (0–5) discrete scale (0: presence of the old feather; 5: fully grown new feather; 1–4: progressively increasing development of the growing feather) (Ginn & Melville, 1983). Molt scoring was performed by very well-trained bird ringers (C S, R L & P M) and was done blind of *Clock* genotype. A total molt score (TMS) was then calculated as the sum of the molt scores of P1-P9 of the right wing, as very high symmetry in the progress of molt between the wings was observed among the first 60 birds that were sampled. Because TMS is computed on nine primaries on a 0–5 scale, its values range between 0 (no growing new feather) and 45 ( = 9 × 5) when all 9 primaries have been molted.

Age (yearling vs. older) was not considered because several yearlings could have completed molt and were therefore indistinguishable from older individuals. However, we can see no reason to speculate that our sampling protocol resulted in different odds of sampling individuals with a different *Clock* genotype according to their age. Before releasing the swallows, we collected a small (ca. 40 µl) blood sample for molecular sexing and *Clock* genotyping purposes. Molecular sexing based on the *CHD* gene and *Clock* genotyping were done according to protocols that we published and were replicated here (Saino, Martinelli & Romano, 2008; Caprioli et al., 2012).

We carefully scrutinized our sampling approach in search of potential sources of bias and, in particular, we considered the possibility that pre-migratory movements, whereby particular classes of individuals (e.g. those that had completed molt) left the roost before we did our sampling, could bias the results. Obviously, because the roost included hundreds of thousands of swallows, we could not monitor all the individuals and how the composition of the roost changed, if it did. However, until February, the roost has constant size (P Micheloni, unpublished data), which then increases in March when barn swallows wintering farther south stop in the area before resuming migration and cross the Sahara. Importantly, the roost is very close (ca. 2° in latitude corresponding to ca. 200 km) to the northern limit of the wintering range of barn swallows, as identified based on all the information on ring recoveries of wintering barn swallows in Africa available to date in the EURING database and encompassing several decades (see Ambrosini et al., 2011). Moreover, recently acquired information from geolocators suggests that during wintering, including February when the present study was conducted, adult (i.e. more than 1-year old) barn swallows, which are the first to complete molt (Møller et al., 2011), are sedentary. Hence, in February barn swallows do not occur north of the region where the study was conducted and do not show pre-migratory movements. Moreover, during February the roost is known to retain constant size (see also Methods), suggesting no “leakage” of individuals or that individuals that leave the roost are replaced by newcomers (but see above) that have also completed molt and should therefore not bias the sample. Other arguments (including e.g. similarity of the proportions of male and females that had or, respectively, had not completed molt) that corroborate the idea that sampling did not bias the results are presented in Saino et al. (2013).

### Analysis of *Clock* gene

Total genomic DNA was extracted by alkaline lysis from blood samples collected in heparinized capillary tubes after puncturing the brachial vein (see Caprioli et al., 2012 for details). Species-specific primers for genotyping purposes were designed initially using the primers reported in Johnsen et al. (2007). The amplified PCR fragments were then sequenced (BMR genomics, Padua, Italy) and a new set of primers were designed on barn swallow genomic sequence. Genomic DNA samples were screened for length polymorphism in the *Clk*polyQcds by PCR amplification followed by resolution and detection on a conventional DNA sequencing machine using a commercial GeneScan service for genotyping (Macrogen Inc., Seoul, Korea) (see Caprioli et al., 2012). We used PCR forward primer (5′-labelled with 6-FAM dye) 5′-[6FAM]GGGACAGGTGGTGACAGCTTATC-3′ and reverse primer 5′-CTGCTGATGGTCCTGCTGACT-3′ (Sigma-Aldrich). Two internal size standards were used in PCR (see Caprioli et al., 2012). The reliability of molecular data were confirmed by randomly picking 80 samples and repeating the GeneScan analysis independently. Moreover each heterozygous genotype was double checked by repeating the PCR amplification reaction and sequencing. The alleles were named according to the number of glutamine residues predicted in the mature protein, as *Q*_6_–*Q*_8_ (Dor et al., 2012). Statistical tests were conducted using genotype classes, i.e. considering *Q*_6_/*Q*_7_, *Q*_7_/*Q*_7_ and *Q*_7_/*Q*_8_ as distinct groups.

### Statistical analyses

We mainly relied on non-parametric tests because of right-truncation and heteroskedasticity of the distribution of molt scores within sexes or genotypes. TMS values were compared between sexes or genotypes using the Mann-Whitney U test. Exact, rather than asymptotic probabilities were always calculated, as these are more appropriate particularly under highly unbalanced designs as in the comparisons of molt scores among genotypes. Tests were performed on the whole sample of sexed or genotyped individuals and then repeated on the sample of individuals that were still actively molting their primaries (i.e. that had TMS ≤ 44; hereafter “molting individuals”) to test for any potential confounding effect of selection of the sampled individuals based on the 3/4–1/4 rule illustrated above. However, the two sets of analyses invariably led to strictly similar results. In significance tests, the α-value was always set at *P* ≤ 0.05. SPSS 13.0 statistical package was used to run all the analyses.

### Ethics statement

All field procedures, including capture, handling and blood sampling were carried out according to standard procedures in this kind of study by expert scientists. Upon capture, swallows were kept in cloth bags in a safe position, as is standard practice in bird ringing studies. Blood samples were collected by puncturing the brachial vein and the puncturing site was disinfected. Individuals were kept overnight in a safe place and released at sunrise the next morning. The study was carried out under invitation by the local authority (Cross River State, Nigeria, TB #1/20/2012).

## Results

### Sex-related variation in molt

Total molt score (TMS) ranged between 22 and 45 in males (median, interquartile range: 37, 10; *n* = 138) and between 18 and 45 in females (39, 10; *n* = 128), implying that all individuals had started molt (see Saino et al., 2013). TMS did not differ between males and females either in the whole sample or in the set of 105 males and 96 females that were still actively molting primaries (Mann-Whitney U test; whole sample: *Z* = 0.88, *P* = 0.382; still molting individuals: *Z* = 1.09, *P* = 0.276). The proportion of males (males/total) in the groups of individuals that were still molting (105/201 = 0.52) or had completed molt (33/65 = 0.51) was closely similar (${\chi }_{1}^{2}=0.84$, *P* = 0.887), again indicating no difference in molt stage between the sexes in the present sample.

We genotyped 282 individuals (146 males, 135 females; sex of one individual could not be assigned), though molt was scored for a subsample of 267 of these genotyped individuals (see also below). Similarly to several breeding populations of barn swallows (Dor et al., 2011; Caprioli et al., 2012), among the swallows wintering in the Boje area *Clock* polymorphism was low, with the most common allele (*Q*_7_) accounting for 96.3% of the total allelic diversity, and allele frequencies were very similar in the two sexes (Table 1). *Q*_7_/*Q*_7_ was by far the most frequent among the three genotypes (*Q*_6_/*Q*_7_, *Q*_7_/*Q*_7_, *Q*_7_/*Q*_8_) that were recorded (Table 1).

**Table 1:** Absolute and relative (%, in parentheses) *Clock* gene frequencies of barn swallows wintering at Boje (Nigeria). *N*: number of alleles or genotypes. One individual included in the overall sample could not be sexed.

	Overall	Males	Females
Alleles			
*Q*_6_	18 (3.2)	10 (3.4)	8 (3.0)
*Q*_7_	543 (96.3)	281 (96.2)	260 (96.3)
*Q*_8_	3 (0.5)	1 (0.3)	2 (0.7)
*N*	564	292	270
Genotypes			
*Q*_6_/*Q*_7_	18 (6.4)	10 (6.8)	8 (5.9)
*Q*_7_/*Q*_7_	261 (92.6)	135 (92.5)	125 (92.6)
*Q*_7_/*Q*_8_	3 (1.1)	1 (0.7)	2 (1.5)
*N*	282	146	135

**Figure 1:**
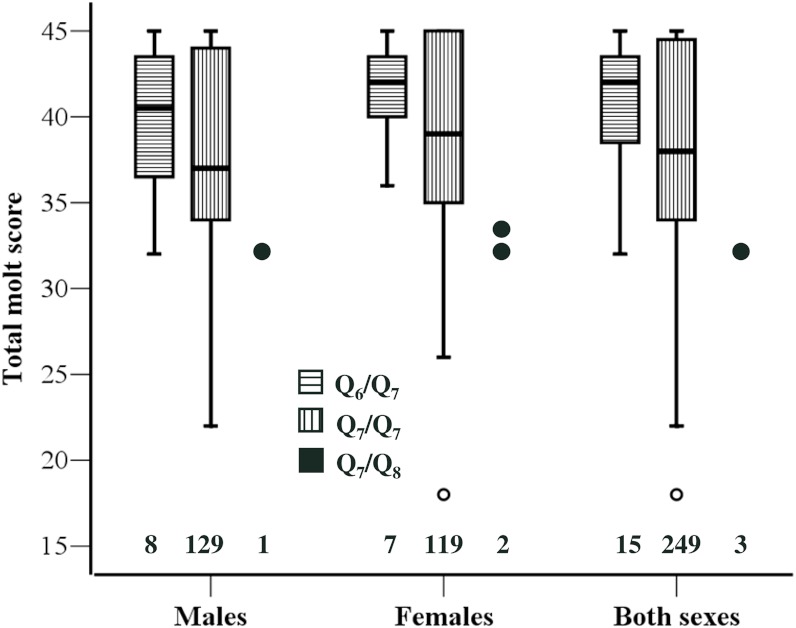
Molt stage of the individuals according to *Clock* genotype. Boxplot of total molt scores for the three *Clock* genotypes. Data for the two sexes separately and for the overall sample are presented. The 25th and 75th percentile (box limits) and the median are shown. Whiskers extend to the smallest and largest values recorded. One extremely low value for *Q*_7_/*Q*_7_ is shown individually. The three data points for *Q*_7_/*Q*_8_ are also presented individually. Sample sizes for each genotype are shown above the *x*-axis. One *Q*_7_/*Q*_7_ individual could not be sexed.

Based on previous findings on the association between *Clock* genotype and timing of breeding (Caprioli et al., 2012), we expected *Q*_7_/*Q*_8_ individuals to exhibit delayed molt compared to *Q*_6_/*Q*_7_ and *Q*_7_/*Q*_7_ ones. Consistently with this prediction we found that TMS of *Q*_7_/*Q*_8_ (*n* = 3) individuals was significantly smaller than that of *Q*_6_/*Q*_7_ (*n* = 15) (*Z* = 2.45, *P* = 0.010) or *Q*_7_/*Q*_7_ (*n* = 249) (*Z* = 2.01, *P* = 0.036) individuals. In this analysis the two sexes were pooled because there was no sex-related variation in TMS. However, there was no significant difference in TMS between *Q*_6_/*Q*_7_ (*n* = 15) and *Q*_7_/*Q*_7_ individuals (*n* = 249; *Z* = 1.15, *P* = 0.248) (Fig. 1).

A similar pattern emerged when the analysis was restricted to individuals that were still molting their primaries, although the difference in TMS was significant between *Q*_7_/*Q*_8_ and *Q*_6_/*Q*_7_ (*Z* = 2.33, *P* = 0.018) but not between *Q*_7_/*Q*_8_ and *Q*_7_/*Q*_7_ (*Z* = 1.67, *P* = 0.100). An analysis restricted to P7, for which we have information on mean growth rates as estimated by ptilochronological analysis (Saino et al., 2012; Saino et al., 2013) confirmed a significant delay of *Q*_7_/*Q*_8_ compared to *Q*_6_/*Q*_7_ (*Z* = 2.99, *P* = 0.005) or *Q*_7_/*Q*_7_ (*Z* = 2.39, *P* = 0.017). Because the difference in P7 molt score between *Q*_7_/*Q*_8_ and the other genotypes was 2.0, P7 are on average 91 mm long (N Saino & D Rubolini, unpublished data), and mean daily growth rate of P7 as estimated by ptilochronology is ca. 4 mm/day (Saino et al., 2012), the difference in molt of P7 between *Q*_7_/*Q*_8_ and the other genotypes can be roughly estimated to correspond to 8–9 days.

The patterns of variation in TMS in relation to *Clock* genotype were strictly similar in the two sexes. The difference between *Q*_6_/*Q*_7_ and *Q*_7_/*Q*_7_ was similar in males and females and far from statistical significance (|*Z*| < 0.95, *P* > 0.356 in both sexes; see Fig. 1). No formal statistical test was conducted on the difference between *Q*_7_/*Q*_8_ and *Q*_6_/*Q*_7_ or *Q*_7_/*Q*_7_ in either sex separately, owing to small number of *Q*_7_/*Q*_8_ heterozygotes particularly among males. However, TMS of *Q*_7_/*Q*_8_ males and females were very similar, as molt score of the single male was 32 while molt scores of the two females were 32 and 33, respectively (Fig. 1).

## Discussion

Our major finding was that rare *Q*_7_/*Q*_8_ heterozygous barn swallows at the *Clock* gene delayed molt relative to the other genotypes. This result emerged despite very small size of the sample of *Q*_7_/*Q*_8_ that was obtained from an otherwise large sample of birds.

In a companion study we showed that timing of molt as measured at four plumage regions did not differ between males and females (Saino et al., 2013) and this finding was confirmed in the present analyses where we focused on primary wing feathers only and that were based on a slightly different sample. We carefully considered potential sources of bias arising from the conditions under which sampling was conducted. Several arguments against potential confounding effects arising from sex-related, pre-migratory movements that might have occurred by mid-February, well before the start of migration, are presented in the Methods as well as in Saino et al. (2013). As far as the evidence of delayed molt by *Q*_7_/*Q*_8_ is specifically concerned, a bias could have arisen from sampling effects only if all of the following conditions were simultaneously met: (1) *Q*_7_/*Q*_8_ individuals had markedly bimodal timing of molt whereby some (a sample of which we could capture) delay molt while others complete molt very early, so that mean molt schedules is similar to that of the other genotypes, and no *Q*_7_/*Q*_8_ individuals with intermediate molt phenotype occur; (2) pre-migratory movements occur already by mid-February and result in a non-negligible proportion of birds leaving the roost (but see above); and (3) *Q*_7_/*Q*_8_ individuals were more likely to leave the roost than the other genotypes. Condition (1) and (3) are obviously very unlikely as it is difficult to imagine how such a sharply bimodal frequency distribution of molt phenotypes could arise and be associated with *Clock* genotype and why such a differential proneness to leave that particular roost should exist among *Clock* genotypes. Condition (2) is also unlikely to be met according to the arguments we presented in the Methods (see also Saino et al., 2013). Hence, we are convinced that sampling effects did not confound the present results.

Although photoperiod is deemed the major extrinsic factor ultimately controlling seasonal activities of birds, including molt (Gwinner, 1986; Dawson et al., 2001), no previous study had investigated variation in molt schedules in relation to polymorphism at genes for photoperiodic response. In barn swallows, the single annual molt of wing and tail feathers normally occurs in sub-Saharan Africa, at tropical latitudes (Cramp, 1998; van den Brink, Bijlsma & van der Have, 2000; Altwegg et al., 2012). Although the role of photoperiod in controlling molt in the tropics may be questioned, birds at the tropics have been shown to respond physiologically to even minor changes in photoperiod (Hau, Wikelski & Wingfield, 1998), and direct evidence for a control of molt and gonadal rhythms by daytime length at low latitudes has been already provided (Gwinner & Scheuerlein, 1998). In addition, it has been recently shown that synchronization of circannual rhythms at the tropics can be achieved by change in time of sunrise and sunset (Goymann et al., 2012). Thus, molt may also be regulated by response to photoperiod and/or daylight intensity variation in barn swallows wintering at the tropics (Gwinner, 2003). Present findings may thus hint at an involvement of *Clock* genes in decoding photoperiodic/daylight intensity information *at the time of molt* and thus in giving a cadence to its progress. A potentially concomitant mechanism is that *Clock* affects timing of other photoperiodic activities that occur earlier at higher latitudes, such as reproduction and initiation of autumn migration. Because of time constraints, these effects may then carry-over on timing of arrival to the wintering grounds and winter molt.

Remarkably, *Q*_7_/*Q*_8_ individuals not only molted later but were also found to delay breeding in a previous study of an Italian population (Caprioli et al., 2012). In fact, delay in breeding was observed only among females, as expected because in birds females have ample control over timing of breeding (Ball & Ketterson, 2008; Liedvogel et al., 2009). The delay in molt by *Q*_7_/*Q*_8_ individuals that could be estimated here based on ptilochronological information on P7 (ca. 8–9 days) is similar in magnitude to the delay that was observed in breeding date of *Q*_7_/*Q*_8_ females compared to the other genotypes (ca. 13 days).

Hence, *Q*_7_/*Q*_8_ is apparently associated with a behavioural syndrome that entails delay in both breeding and molt. The cascade of causation between *Clock*, and breeding and molt phenology, however, is uncertain: owing to tight annual routines, delayed breeding may cause molt retardation or, conversely, delayed molt may cause late migration and thus breeding. Finally, *Clock* genotype may have an independent effect on both timing of breeding and molt.

In theory, the association between *Clock* and molt could be due to a second gene linked to *Clock*. However, the large body of evidence showing *Clock* involvement in photoperiodic responses in several, phylogenetically distant organisms (see above) which are unlikely to share syntenic chromosomes makes this hypothesis unlikely. A partly alternative interpretation of the present findings is that *Clock* has an indirect role in controlling molt by determining circadian activity rhythms, including for example foraging behaviour.

Barn swallows from diverse western and central European regions converge to winter in Nigeria, and in the Boje area in particular, as shown by analysis of ring recoveries (P Micheloni, unpublished data). Hypothetically, a spurious association between molt scores and *Clock* genotype could result from latitudinal variation in both *Clock* allele frequencies and molt schedules. This interpretation, however, is very unlikely to be correct given the lack of geographical variation in *Clock* gene frequencies that emerges from two studies of six distant geographic populations and from unpublished information on an additional northern European population (Dor et al., 2011; Caprioli et al., 2012; N Saino, M Caprioli & AP Møller, unpublished data).

Finally, a different interpretation is that our results are driven by selection against *Q*_7_/*Q*_8_. In a previous study it has been demonstrated that the frequency of *Q*_7_/*Q*_8_ is larger among yearlings than older individuals (Caprioli et al., 2012). Because yearlings molt later, it is possible that delayed molt of *Q*_7_/*Q*_8_ results from a larger frequency of *Q*_7_/*Q*_8_ in the yearlings fraction of the population. In this perspective, the present findings suggest that delayed molt may concur in determining reduced viability of rare *Q*_7_/*Q*_8_ individuals. This interpretation is also unlikely to be correct, however, because we observed the same pattern of differences in molt scores among genotypes when we considered the subsample of individuals that were still actively molting their wing feathers, which was likely formed mainly by yearlings (Møller et al., 2011).

Admittedly, the present results have little specific bearing for the study of molt phenology of barn swallow populations, because *Q*_8_ has been found to be very rare in all populations studied so far. Conversely, the present findings have a potentially major relevance for studies of genetic control of molt and, more generally, for studies of photoperiodism and regulation of seasonality in birds, including the studies on phenotypically plastic/microevolutionary phenological responses to current climate change (see Knudsen et al., 2011 for a review). While low frequency of *Q*_8_ in our model population may be maintained by selection (Caprioli et al., 2012), higher polymorphism at *Clock* in other species (Johnsen et al., 2007; Liedvogel & Sheldon, 2010; Dor et al., 2012) may be maintained by selection for variation in photoperiodic response strategies due to e.g. spatial or temporal variation in selection pressures, therefore affording ample opportunity to study the effect of *Clock* on timing of molt.

## Conclusion

Using molecular techniques to identify sex, this study of barn swallows achieved a novel finding of potentially general relevance for the study of the evolution and the control of molt and migration strategies in birds, and potentially in other organisms where the *Clock* genetic machinery of control of photoperiodic responses is conserved. We uncovered an association between timing of molt and *Clock* genotype and found that rare *Q*_7_/*Q*_8_ individuals, which are known to delay breeding, also show delayed annual molt in winter. The pleiotropic temporal shift that *Q*_7_/*Q*_8_ may cause in fundamental life-history components has obvious general implications for the understanding of regulation of seasonality and annual routines particularly in long-distance migratory species with complex annual cycles, and might disclose research avenues particularly in species exhibiting larger polymorphism at *Clock* genes.

## Additional Information and Declarations

### Field Study Permissions

The following information was supplied relating to ethical approvals (i.e. approving body and any reference numbers): The study was carried out under invitation by the local authority (Cross River State, Nigeria, TB #1/20/2012).
